# *N*′-(3-Bromo-5-chloro-2-hydroxy­benzyl­idene)-3,4,5-trihydroxy­benzohydrazide methanol solvate

**DOI:** 10.1107/S1600536809010575

**Published:** 2009-03-28

**Authors:** Abeer A. Abdul Alhadi, Hapipah Mohd. Ali, Seik Weng Ng

**Affiliations:** a Department of Chemistry, University of Malaya, 50603 Kuala Lumpur, Malaysia

## Abstract

The benzohydrazide mol­ecule of the title compound, C_14_H_10_BrClN_2_O_5_·CH_3_OH, is non-planar, the two aromatic rings at either side of the –C(=O)–NH–N=CH– unit being twisted by 5.9 (1)°. The benzohydrazide mol­ecule is linked to the solvent mol­ecule by an O—H⋯O hydrogen bond. Mol­ecules are connected by further O—H⋯O hydrogen bonds and an N—H⋯O link into a two-dimensional array.

## Related literature

For the the parent *N*′-(2-hydroxy­benzyl­idene)benzohydrazide, see: Lyubchova *et al.* (1995 ▶). For other *N*′-(2-hydr­oxy-5-nitro­benzyl­idene)benzohydrazides, see: Ali *et al.* (2005 ▶); Lyubchova *et al.* (1995 ▶); Xu & Liu (2006 ▶).
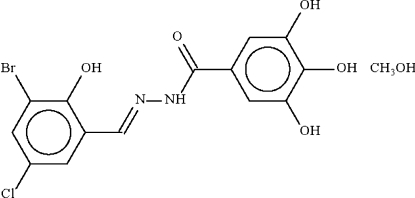


## Experimental

### 

#### Crystal data

C_14_H_10_BrClN_2_O_5_·CH_4_O
*M* *_r_* = 433.64
Monoclinic, 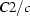

*a* = 21.6157 (3) Å
*b* = 12.7408 (2) Å
*c* = 17.0803 (2) Å
β = 136.641 (1)°
*V* = 3229.57 (8) Å^3^
*Z* = 8
Mo *K*α radiation
μ = 2.75 mm^−1^
*T* = 123 K
0.20 × 0.15 × 0.10 mm

#### Data collection

Bruker SMART APEX diffractometer
Absorption correction: multi-scan (*SADABS*; Sheldrick, 1996 ▶) *T*_min_ = 0.610, *T*_max_ = 0.771
15301 measured reflections
3714 independent reflections
3407 reflections with *I* > 2σ(*I*)
*R*_int_ = 0.016

#### Refinement

*R*[*F* ^2^ > 2σ(*F* ^2^)] = 0.023
*wR*(*F* ^2^) = 0.068
*S* = 1.03
3714 reflections
251 parameters
6 restraints
H atoms treated by a mixture of independent and constrained refinement
Δρ_max_ = 0.43 e Å^−3^
Δρ_min_ = −0.35 e Å^−3^

### 

Data collection: *APEX2* (Bruker, 2008 ▶); cell refinement: *SAINT* (Bruker, 2008 ▶); data reduction: *SAINT*; program(s) used to solve structure: *SHELXS97* (Sheldrick, 2008 ▶); program(s) used to refine structure: *SHELXL97* (Sheldrick, 2008 ▶); molecular graphics: *X-SEED* (Barbour, 2001 ▶); software used to prepare material for publication: *publCIF* (Westrip, 2009 ▶).

## Supplementary Material

Crystal structure: contains datablocks global, I. DOI: 10.1107/S1600536809010575/tk2400sup1.cif

Structure factors: contains datablocks I. DOI: 10.1107/S1600536809010575/tk2400Isup2.hkl

Additional supplementary materials:  crystallographic information; 3D view; checkCIF report

## Figures and Tables

**Table 1:** Hydrogen-bond geometry (Å, °)

*D*—H⋯*A*	*D*—H	H⋯*A*	*D*⋯*A*	*D*—H⋯*A*
O1—H1⋯O5^i^	0.83 (1)	2.03 (3)	2.607 (2)	126 (3)
O3—H3⋯O6^ii^	0.83 (1)	2.05 (1)	2.856 (2)	165 (3)
O4—H4⋯O1^iii^	0.83 (1)	2.20 (1)	3.027 (2)	173 (2)
O5—H5⋯O2^iii^	0.82 (1)	1.79 (1)	2.608 (2)	176 (2)
O6—H6⋯O2	0.83 (1)	2.04 (1)	2.843 (2)	164 (2)
N2—H2⋯O6^iii^	0.87 (1)	2.25 (1)	3.112 (2)	169 (2)

Symmetry codes: (i) 
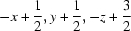
; (ii) 
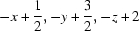
; (iii) 
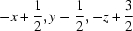
.

**Fig. 1.**
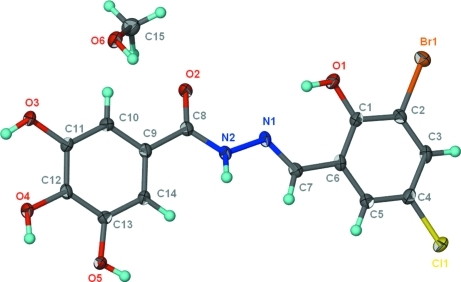
Thermal ellipsoid plot (Barbour, 2001) of C14H10BrClNO5.CH3OH at the 70% probability level; hydrogen atoms are drawn as spheres of arbitrary radius.

## supplementary crystallographic information

### Experimental

3-Bromo-5-chloro-2-hydroxybenzaldehyde (0.47 g, 2 mmol) and 3,4,5-trihydroxybenzoylhydrazide (0.36 g, 2 mmol) were heated in ethanol (50 ml) for several hours. The solvent was removed and the product recrystallized from methanol.

### Refinement

Carbon-bound H-atoms were placed in calculated positions [C—H 0.95 Å, *U*(H) = 1.2*U*(C)], and were included in the refinement in the riding model approximation.

The amino and hydroxy H-atoms were located in a difference Fourier map, and were refined with distance restraints of N–H 0.88±0.01 Å and O–H 0.84±0.01 Å, respectively; their temperature factors were refined isotropically.

### Crystal data

**Table:**

C_14_H_10_BrClN_2_O_5_·CH_4_O	*F*(000) = 1744
*M**_r_* = 433.64	*D*_x_ = 1.784 Mg m^−^^3^
Monoclinic, *C*2/*c*	Mo *K*α radiation, λ = 0.71073 Å
Hall symbol: -C 2yc	Cell parameters from 8789 reflections
*a* = 21.6157 (3) Å	θ = 2.7–28.3°
*b* = 12.7408 (2) Å	µ = 2.75 mm^−^^1^
*c* = 17.0803 (2) Å	*T* = 123 K
β = 136.641 (1)°	Polyhedral, tan
*V* = 3229.57 (8) Å^3^	0.20 × 0.15 × 0.10 mm
*Z* = 8	

### Data collection

**Table:**

Bruker SMART APEX diffractometer	3714 independent reflections
Radiation source: fine-focus sealed tube	3407 reflections with *I* > 2σ(*I*)
graphite	*R*_int_ = 0.016
ω scans	θ_max_ = 27.5°, θ_min_ = 2.0°
Absorption correction: multi-scan (*SADABS*; Sheldrick, 1996)	*h* = −28→26
*T*_min_ = 0.610, *T*_max_ = 0.771	*k* = −16→16
15301 measured reflections	*l* = −22→21

### Refinement

**Table:**

Refinement on *F*^2^	Primary atom site location: structure-invariant direct methods
Least-squares matrix: full	Secondary atom site location: difference Fourier map
*R*[*F*^2^ > 2σ(*F*^2^)] = 0.023	Hydrogen site location: inferred from neighbouring sites
*wR*(*F*^2^) = 0.068	H atoms treated by a mixture of independent and constrained refinement
*S* = 1.03	*w* = 1/[σ^2^(*F*_o_^2^) + (0.0429*P*)^2^ + 3.4447*P*] where *P* = (*F*_o_^2^ + 2*F*_c_^2^)/3
3714 reflections	(Δ/σ)_max_ = 0.001
251 parameters	Δρ_max_ = 0.43 e Å^−^^3^
6 restraints	Δρ_min_ = −0.35 e Å^−^^3^

### Fractional atomic coordinates and isotropic or equivalent isotropic displacement parameters (Å^2^)

**Table:**

	*x*	*y*	*z*	*U*_iso_*/*U*_eq_	
Br1	0.044783 (12)	0.783092 (13)	0.137916 (14)	0.02172 (7)	
Cl1	0.01420 (3)	0.35175 (3)	0.10946 (4)	0.02369 (10)	
N1	0.18779 (9)	0.61375 (11)	0.54542 (11)	0.0148 (3)	
N2	0.23179 (9)	0.58418 (11)	0.65407 (11)	0.0154 (3)	
O1	0.13116 (9)	0.74001 (9)	0.37133 (11)	0.0211 (3)	
O2	0.20939 (8)	0.74133 (9)	0.69379 (10)	0.0187 (2)	
O3	0.36037 (9)	0.69205 (10)	1.09583 (10)	0.0219 (3)	
O4	0.37603 (8)	0.47725 (9)	1.12740 (10)	0.0189 (2)	
O5	0.31633 (9)	0.34724 (9)	0.95863 (10)	0.0205 (3)	
O6	0.15053 (8)	0.88367 (9)	0.75713 (10)	0.0196 (2)	
C1	0.10878 (11)	0.64859 (12)	0.31698 (13)	0.0155 (3)	
C2	0.06644 (11)	0.65171 (12)	0.20499 (14)	0.0156 (3)	
C3	0.03858 (11)	0.56163 (13)	0.14173 (13)	0.0172 (3)	
H3A	0.0088	0.5655	0.0652	0.021*	
C4	0.05469 (11)	0.46540 (12)	0.19171 (14)	0.0168 (3)	
C5	0.09940 (10)	0.45814 (12)	0.30364 (13)	0.0158 (3)	
H5A	0.1117	0.3914	0.3373	0.019*	
C6	0.12644 (10)	0.54979 (12)	0.36718 (13)	0.0151 (3)	
C7	0.17192 (10)	0.53678 (12)	0.48399 (13)	0.0155 (3)	
H7	0.1907	0.4684	0.5163	0.019*	
C8	0.23645 (10)	0.64897 (12)	0.72065 (13)	0.0147 (3)	
C9	0.27436 (10)	0.60244 (12)	0.82779 (13)	0.0143 (3)	
C10	0.30257 (11)	0.66835 (12)	0.91445 (13)	0.0161 (3)	
H10	0.2997	0.7424	0.9056	0.019*	
C11	0.33473 (11)	0.62583 (12)	1.01356 (13)	0.0156 (3)	
C12	0.34227 (10)	0.51690 (12)	1.02835 (13)	0.0146 (3)	
C13	0.31234 (10)	0.45163 (12)	0.94055 (13)	0.0147 (3)	
C14	0.27745 (10)	0.49359 (12)	0.84020 (13)	0.0146 (3)	
H14	0.2557	0.4486	0.7800	0.018*	
C15	0.06333 (12)	0.92150 (14)	0.65359 (15)	0.0237 (4)	
H15A	0.0445	0.9781	0.6715	0.036*	
H15B	0.0196	0.8640	0.6160	0.036*	
H15C	0.0664	0.9482	0.6027	0.036*	
H1	0.154 (2)	0.731 (3)	0.4365 (15)	0.087 (13)*	
H3	0.3635 (18)	0.6613 (19)	1.1413 (18)	0.044 (7)*	
H4	0.3783 (17)	0.4119 (8)	1.129 (2)	0.036 (7)*	
H5	0.3065 (15)	0.3125 (16)	0.9101 (15)	0.030 (6)*	
H6	0.1634 (16)	0.8331 (14)	0.741 (2)	0.037 (7)*	
H2	0.2604 (14)	0.5242 (11)	0.6799 (18)	0.029 (6)*	

### Atomic displacement parameters (Å^2^)

**Table:**

	*U*^11^	*U*^22^	*U*^33^	*U*^12^	*U*^13^	*U*^23^
Br1	0.02904 (11)	0.01747 (10)	0.01845 (10)	0.00031 (6)	0.01720 (9)	0.00285 (6)
Cl1	0.0373 (2)	0.01580 (19)	0.01895 (19)	−0.00382 (16)	0.02079 (19)	−0.00522 (14)
N1	0.0169 (6)	0.0172 (6)	0.0109 (6)	0.0006 (5)	0.0103 (5)	0.0013 (5)
N2	0.0205 (6)	0.0142 (6)	0.0119 (6)	0.0032 (5)	0.0120 (6)	0.0031 (5)
O1	0.0321 (7)	0.0152 (5)	0.0161 (6)	0.0006 (5)	0.0175 (6)	−0.0016 (5)
O2	0.0293 (6)	0.0135 (5)	0.0186 (6)	0.0039 (5)	0.0191 (5)	0.0034 (4)
O3	0.0374 (7)	0.0154 (5)	0.0171 (6)	−0.0039 (5)	0.0212 (6)	−0.0038 (5)
O4	0.0294 (6)	0.0154 (6)	0.0142 (5)	0.0022 (5)	0.0166 (5)	0.0019 (4)
O5	0.0378 (7)	0.0109 (5)	0.0182 (6)	0.0028 (5)	0.0221 (6)	0.0017 (4)
O6	0.0251 (6)	0.0178 (6)	0.0186 (6)	0.0016 (5)	0.0168 (5)	−0.0013 (5)
C1	0.0184 (7)	0.0151 (7)	0.0154 (7)	−0.0006 (6)	0.0131 (7)	−0.0020 (6)
C2	0.0186 (7)	0.0153 (7)	0.0153 (7)	0.0015 (6)	0.0130 (7)	0.0026 (6)
C3	0.0185 (7)	0.0226 (8)	0.0135 (7)	−0.0012 (6)	0.0126 (7)	−0.0011 (6)
C4	0.0202 (7)	0.0170 (7)	0.0169 (7)	−0.0017 (6)	0.0147 (7)	−0.0037 (6)
C5	0.0184 (7)	0.0153 (7)	0.0164 (7)	0.0005 (6)	0.0136 (6)	0.0007 (6)
C6	0.0164 (7)	0.0182 (7)	0.0129 (7)	0.0004 (6)	0.0113 (6)	0.0003 (6)
C7	0.0173 (7)	0.0156 (7)	0.0135 (7)	0.0006 (6)	0.0112 (6)	0.0017 (6)
C8	0.0165 (7)	0.0151 (7)	0.0138 (7)	−0.0016 (6)	0.0114 (6)	−0.0007 (5)
C9	0.0158 (7)	0.0158 (7)	0.0126 (7)	0.0010 (6)	0.0107 (6)	0.0009 (6)
C10	0.0212 (8)	0.0123 (7)	0.0166 (8)	−0.0005 (6)	0.0144 (7)	−0.0003 (6)
C11	0.0198 (7)	0.0148 (7)	0.0140 (7)	−0.0021 (6)	0.0129 (6)	−0.0030 (6)
C12	0.0168 (7)	0.0167 (7)	0.0123 (7)	0.0009 (6)	0.0112 (6)	0.0013 (6)
C13	0.0180 (7)	0.0121 (7)	0.0168 (7)	0.0016 (6)	0.0136 (6)	0.0006 (6)
C14	0.0189 (7)	0.0136 (7)	0.0144 (7)	0.0005 (6)	0.0131 (6)	−0.0005 (5)
C15	0.0247 (8)	0.0219 (8)	0.0214 (8)	0.0000 (7)	0.0158 (7)	−0.0015 (7)

### Geometric parameters (Å, °)

**Table:**

Br1—C2	1.8852 (15)	C3—C4	1.386 (2)
Cl1—C4	1.7460 (16)	C3—H3A	0.9500
N1—C7	1.287 (2)	C4—C5	1.382 (2)
N1—N2	1.3870 (18)	C5—C6	1.400 (2)
N2—C8	1.348 (2)	C5—H5A	0.9500
N2—H2	0.874 (10)	C6—C7	1.457 (2)
O1—C1	1.3415 (19)	C7—H7	0.9500
O1—H1	0.832 (10)	C8—C9	1.480 (2)
O2—C8	1.2438 (19)	C9—C14	1.397 (2)
O3—C11	1.3642 (19)	C9—C10	1.398 (2)
O3—H3	0.828 (10)	C10—C11	1.390 (2)
O4—C12	1.3619 (18)	C10—H10	0.9500
O4—H4	0.833 (10)	C11—C12	1.399 (2)
O5—C13	1.3538 (18)	C12—C13	1.396 (2)
O5—H5	0.820 (10)	C13—C14	1.383 (2)
O6—C15	1.437 (2)	C14—H14	0.9500
O6—H6	0.828 (10)	C15—H15A	0.9800
C1—C2	1.397 (2)	C15—H15B	0.9800
C1—C6	1.410 (2)	C15—H15C	0.9800
C2—C3	1.379 (2)		
			
C7—N1—N2	113.63 (13)	C6—C7—H7	118.5
C8—N2—N1	121.35 (13)	O2—C8—N2	122.77 (14)
C8—N2—H2	121.2 (15)	O2—C8—C9	121.61 (14)
N1—N2—H2	117.2 (15)	N2—C8—C9	115.61 (13)
C1—O1—H1	112 (3)	C14—C9—C10	119.97 (14)
C11—O3—H3	111.6 (19)	C14—C9—C8	120.49 (14)
C12—O4—H4	112.6 (18)	C10—C9—C8	119.44 (14)
C13—O5—H5	111.9 (17)	C11—C10—C9	120.15 (14)
C15—O6—H6	107.8 (17)	C11—C10—H10	119.9
O1—C1—C2	118.05 (14)	C9—C10—H10	119.9
O1—C1—C6	123.52 (14)	O3—C11—C10	118.85 (14)
C2—C1—C6	118.42 (14)	O3—C11—C12	121.39 (14)
C3—C2—C1	121.82 (14)	C10—C11—C12	119.75 (14)
C3—C2—Br1	119.27 (12)	O4—C12—C13	121.65 (14)
C1—C2—Br1	118.90 (12)	O4—C12—C11	118.63 (14)
C2—C3—C4	118.86 (14)	C13—C12—C11	119.67 (14)
C2—C3—H3A	120.6	O5—C13—C14	123.32 (14)
C4—C3—H3A	120.6	O5—C13—C12	115.94 (14)
C5—C4—C3	121.40 (14)	C14—C13—C12	120.69 (14)
C5—C4—Cl1	119.65 (12)	C13—C14—C9	119.64 (14)
C3—C4—Cl1	118.92 (12)	C13—C14—H14	120.2
C4—C5—C6	119.62 (14)	C9—C14—H14	120.2
C4—C5—H5A	120.2	O6—C15—H15A	109.5
C6—C5—H5A	120.2	O6—C15—H15B	109.5
C5—C6—C1	119.81 (14)	H15A—C15—H15B	109.5
C5—C6—C7	116.89 (14)	O6—C15—H15C	109.5
C1—C6—C7	123.30 (14)	H15A—C15—H15C	109.5
N1—C7—C6	122.97 (14)	H15B—C15—H15C	109.5
N1—C7—H7	118.5		
			
C7—N1—N2—C8	−165.52 (15)	N1—N2—C8—C9	172.04 (14)
O1—C1—C2—C3	−177.88 (15)	O2—C8—C9—C14	159.38 (15)
C6—C1—C2—C3	2.7 (2)	N2—C8—C9—C14	−19.4 (2)
O1—C1—C2—Br1	0.8 (2)	O2—C8—C9—C10	−17.1 (2)
C6—C1—C2—Br1	−178.63 (11)	N2—C8—C9—C10	164.18 (15)
C1—C2—C3—C4	−1.2 (2)	C14—C9—C10—C11	0.8 (2)
Br1—C2—C3—C4	−179.92 (12)	C8—C9—C10—C11	177.25 (14)
C2—C3—C4—C5	−1.3 (2)	C9—C10—C11—O3	−178.92 (15)
C2—C3—C4—Cl1	176.64 (12)	C9—C10—C11—C12	2.6 (2)
C3—C4—C5—C6	2.2 (2)	O3—C11—C12—O4	0.0 (2)
Cl1—C4—C5—C6	−175.66 (12)	C10—C11—C12—O4	178.42 (14)
C4—C5—C6—C1	−0.7 (2)	O3—C11—C12—C13	177.72 (14)
C4—C5—C6—C7	179.02 (14)	C10—C11—C12—C13	−3.9 (2)
O1—C1—C6—C5	178.92 (15)	O4—C12—C13—O5	2.0 (2)
C2—C1—C6—C5	−1.7 (2)	C11—C12—C13—O5	−175.63 (14)
O1—C1—C6—C7	−0.8 (2)	O4—C12—C13—C14	179.35 (14)
C2—C1—C6—C7	178.61 (14)	C11—C12—C13—C14	1.7 (2)
N2—N1—C7—C6	−179.99 (14)	O5—C13—C14—C9	178.84 (15)
C5—C6—C7—N1	−169.07 (15)	C12—C13—C14—C9	1.7 (2)
C1—C6—C7—N1	10.7 (2)	C10—C9—C14—C13	−3.0 (2)
N1—N2—C8—O2	−6.7 (2)	C8—C9—C14—C13	−179.38 (14)

### Hydrogen-bond geometry (Å, °)

**Table:**

*D*—H···*A*	*D*—H	H···*A*	*D*···*A*	*D*—H···*A*
O1—H1···O5^i^	0.83 (1)	2.03 (3)	2.607 (2)	126 (3)
O3—H3···O6^ii^	0.83 (1)	2.05 (1)	2.856 (2)	165 (3)
O4—H4···O1^iii^	0.83 (1)	2.20 (1)	3.027 (2)	173 (2)
O5—H5···O2^iii^	0.82 (1)	1.79 (1)	2.608 (2)	176 (2)
O6—H6···O2	0.83 (1)	2.04 (1)	2.843 (2)	164 (2)
N2—H2···O6^iii^	0.87 (1)	2.25 (1)	3.112 (2)	169 (2)

Symmetry codes: (i) −*x*+1/2, *y*+1/2, −*z*+3/2; (ii) −*x*+1/2, −*y*+3/2, −*z*+2; (iii) −*x*+1/2, *y*−1/2, −*z*+3/2.
